# Efficacy and tolerability of treatment with azacitidine for 5 days in elderly patients with acute myeloid leukemia

**DOI:** 10.1002/cam4.321

**Published:** 2014-08-16

**Authors:** Santhosh K Sadashiv, Christie Hilton, Cyrus Khan, James M Rossetti, Heather L Benjamin, Salman Fazal, Entezam Sahovic, Richard K Shadduck, John Lister

**Affiliations:** 1 Division of Hematology and Cellular Therapy, Allegheny Health NetworkPittsburgh, Pennsylvania, 15224

**Keywords:** acute myeloid leukemia, aged, Azacitidine, outpatients, subcutaneous injections

## Abstract

Acute myeloid leukemia (AML) patients aged ≥60 years tolerate standard induction chemotherapy poorly. Therapy with azacitidine at a dose of 75 mg/m^2^/day for 7 days appears to be better tolerated, and is approved by the Food and Drug Administration (FDA) for the treatment of elderly AML patients with bone marrow (BM) blast counts of 20–30%. Here, we report the results of a prospective, phase 2, open-label study that evaluated the tolerability and efficacy of a 5-day regimen of single-agent subcutaneous azacitidine 100 mg/m^2^/day administered every 28 days in 15 elderly patients with newly diagnosed AML, 14 of whom had BM blast counts >30%. The overall response rate was 47%. Complete remission, partial remission, and hematologic improvement were achieved by 20, 13, and 13% of patients, respectively. Median overall survival was 355 days for the entire cohort, and 532 days for responders. Median time to best response was 95 days, and median treatment duration was 198 days (range = 13–724 days). Grade 3–4 hematologic toxicities comprised predominantly febrile neutropenia (40%) and thrombocytopenia (20%). Febrile neutropenia was the most common cause of hospitalization. Nonhematologic toxicities, consisting of injection-site skin reactions and fatigue (Grades 1–2), occurred in 73% (*n* = 11) of patients. No treatment-related deaths occurred during the study. The dose and schedule of therapy remained constant in all but four patients. The findings of this study suggest that administration of subcutaneous azacitidine 100 mg/m^2^/day for 5 days every 28 days is a feasible, well-tolerated, and effective alternative to standard induction chemotherapy in elderly patients with AML.

## Introduction

The incidence of acute myeloid leukemia (AML) increases with age, making it predominantly a disease of the elderly, and treatment of this population presents a unique challenge. Newly diagnosed AML in patients aged ≥60 years carries a poor prognosis, regardless of therapy [1]. It is estimated that there were around 14,590 new cases of AML in the United States in 2013, with more than 40% of these individuals aged ≥65 years at the time of diagnosis [2].

Standard induction therapy for AML, except for acute promyelocytic leukemia, combines an anthracycline with cytarabine [3], in an attempt to induce complete remission (CR). If remission is achieved, consolidation therapy is administered to maintain this condition. This strategy is associated with considerable morbidity, even in carefully selected patients [4]. Such morbidity is further amplified in patients aged ≥65 years, in whom induction mortality rates are as high as 29%, while induction of remission is achieved in only 30–50% of this population, compared with >70% of patients aged <60 years [3, 5–8]. In addition to a reduced remission rate, only about 20% of elderly patients are able to achieve a durable remission, regardless of the type of consolidation therapy administered [7]. In a large series of 1005 patients, only 28% of patients with AML who were aged >60 years were still alive 1 year after diagnosis [9].

Although intensive induction chemotherapy improves survival in AML, and should be offered to carefully selected patients, the use of such regimens to treat elderly patients with AML is challenging [10]. The elderly often have comorbid conditions, a decreased performance status [11], a higher incidence of unfavorable cytogenetics [11], and a lower overall response rate (ORR) than younger patients, leading to greater morbidity and mortality with intensive induction therapy [6, 11, 12]. Hence, many patients aged >65 years may not be candidates for intensive chemotherapy regimens [13, 14]. In patients not fit for intensive induction chemotherapy, treatment with low-dose cytarabine has been used, and has shown a modest benefit [15, 16]. In a multicenter, phase 3 study of 217 patients with AML or high-risk myelodysplastic syndromes (MDS), low-dose cytarabine improved CR rates versus hydroxyurea (18% vs. 1%), with a survival benefit limited to patients who achieved a CR (80 weeks vs. 18 weeks) [15]. The overall survival (OS) for the entire cohort was <1% at 3 years, suggesting that the prognosis in patients aged >60 years remains dismal [15].

Recent epigenetic studies have detected abnormal DNA hypermethylation of multiple genes in AML [17]. Azacitidine (VIDAZA®; Celgene Corporation, Summit, NJ), a chemical analog of the pyrimidine nucleoside cytosine, is believed to exert its action through direct incorporation into RNA, leading to disruption of protein synthesis and eventual apoptosis of the abnormal hematopoietic stem cells in the bone marrow (BM) [15]. In addition, as a hypomethylating agent, it incorporates directly into DNA and inhibits DNA methyltransferase, which is known to cause epigenetic silencing of tumor suppressor genes. In experimental models, DNA methyltransferase has been shown to result in tumorigenesis [18].

Based on these findings, alternative, azacitidine-incorporating treatments for AML in elderly patients have been studied. Several trials, including the Cancer and Acute Leukemia Group B (CALGB) 9221 and AZA-001 trials, have established the effectiveness and survival benefit of azacitidine 75 mg/m^2^/day over 7 days in elderly patients with AML, as defined by the World Health Organization [13, 14, 19]. Although these trials were intended to treat patients with MDS, a subset of patients with a BM myeloblast count of 20–30% were reclassified as having AML.

In a prior retrospective analysis carried out at our institution, azacitidine was associated with an ORR of 60% and limited toxicity when administered at a dose of 75 mg/m^2^ per day over 7 days to patients >55 years of age with newly diagnosed AML [20]. In addition, other studies have demonstrated a survival benefit, despite low rates of CR, following treatment with azacitidine [13, 21, 22]. These findings were predominantly in patients with BM blast counts of 20–30%, and limited information is currently available regarding the benefits of azacitidine treatment in elderly patients with BM blast counts >30% [21, 23]. Moreover, the best azacitidine dosing regimen in this particular subset of patients is not clearly defined, as such patients were largely excluded from earlier clinical trials [24].

Here, we describe the outcomes of 15 patients enrolled in an open-label, phase 2 trial (WPCI 2005-19), which was designed primarily to assess the efficacy and tolerability of treatment with azacitidine 100 mg/m^2^/day for 5 days in patients aged ≥60 years with newly diagnosed AML. We hypothesized that, given the outpatient nature of azacitidine treatment, a 5-day regimen would be preferable to a 7-day regimen, while providing similar efficacy.

## Materials and Methods

### Study design

This was a prospective, single-institution, open-label, phase 2 study of the efficacy of azacitidine as induction therapy in elderly patients with newly diagnosed AML. All patients were required to provide informed, written consent, and institutional review board approval was obtained and maintained throughout the study.

The primary objectives were OS and remission rates, where remission rates were defined by the National Cancer Institute-sponsored workshop on AML [25] or International Working Group criteria for MDS in patients who do not meet AML response criteria but demonstrate hematologic response [26].

Secondary objectives included transfusion requirements, time to maximum response, treatment-related morbidity and mortality, Eastern Cooperative Oncology Group performance status (ECOG PS), and number of inpatient hospital days.

### Inclusion/exclusion criteria

Patients aged ≥60 years with newly diagnosed AML, who were deemed poor candidates for induction therapy and had an ECOG PS ≤2, were eligible for inclusion in the study. The diagnosis of AML required a BM blast count ≥20%, with confirmation of myeloid phenotype by flow cytometry and immunohistochemistry. Patients were required to have a peripheral blood blast count ≤30.0 × 10^9^/L at the start of therapy. Those with a peripheral blood blast count >30.0 × 10^9^/L were treated with hydroxyurea until it was ≤30.0 × 10^9^/L. Exclusion criteria included: acute promyelocytic leukemia (AML M3); hypersensitivity to azacitidine or mannitol; advanced malignancy with an estimated survival of <12 months; and prior exposure to investigational agents within 30 days of study enrollment. A prior history of MDS did not exclude participation, provided that the patient had no prior exposure to hypomethylating agents. Patients who received at least one cycle of azacitidine were considered eligible for evaluation of response.

### Treatment regimen

All patients received azacitidine at a dose of 100 mg/m^2^/day for five consecutive days every 28 days via subcutaneous injection. This dose was chosen to provide a cumulative dose that was as close as possible to the cumulative dose delivered by the standard 7-day regimen of azacitidine 75 mg/m^2^/day. The first cycle of therapy began on a Monday, unless the attending physician believed that a delay in therapy would be detrimental. If cycle 1 started on any day other than a Monday, subsequent cycles were adjusted to begin on the following Monday, as directed by the attending physician. Patients received at least three cycles of treatment, unless it was discontinued because of disease progression or toxicity, as determined by the attending physician. If no response was seen prior to the start of cycle 4, the azacitidine dose was increased to 125 mg/m^2^ for at least three cycles, utilizing the same five consecutive days every 28 days dosing strategy. Patients were permitted to delay the next azacitidine cycle for up to 4 weeks to allow recovery from grade 4 toxicity. 5-hydroxytryptamine receptor (5-HT3) antagonists were administered prior to each dose of azacitidine. Patients were permitted to remain on the study drug for as long as their disease state either remained stable or improved, and the therapy was tolerated. Any patient was able to withdraw voluntarily from the study at any time, for any reason.

### Initial evaluation, monitoring, and assessment of response

Patients were evaluated prior to enrollment by means of: medical history; physical examination; assessment of ECOG PS; complete blood count with differential; complete metabolic panel analyses; coagulation parameters; BM aspirate assessments; and a biopsy, including flow cytometry and cytogenetic analysis. Prior to each cycle, patients were assessed by review of medical history, physical assessment (including ECOG PS), complete blood count with differential, and complete metabolic panel analyses. Monitoring during the study included a BM biopsy and aspirate with flow cytometry and cytogenetic analysis prior to the start of every fourth cycle of azacitidine for the first 12 cycles, and then every six cycles.

The following responses were assessed:

1. OS, defined as the time from inclusion to death from any cause
2. CR and partial remission (PR), as defined by the National Cancer Institute (NCI)-sponsored workshop on AML [25]
3. Hematologic improvement (HI) according to the or International Working Group criteria for MDS, if patients did not meet the AML response criteria but demonstrated HI [26]
4. Relapse, defined as the reappearance of blasts in the blood or the finding of >5% blasts in the BM, not attributable to any other cause (e.g., BM regeneration) [26]
5. Progression/relapse after HI, defined as meeting one or more of the following criteria: >50% decline from best response levels in granulocytes or platelets; reduction in hemoglobin concentration >2 g/dL; or transfusion dependence in the absence of any other explanation [26].

### Supportive care

Antimicrobial prophylaxis with quinolone antibiotic and fluconazole were given to patients during periods of severe neutropenia (absolute neutrophil count [ANC] <500 cells/*μ*L). Growth factor support, in the form of pegfilgrastim or filgrastim, was permitted for ANC <500 cells/*μ*L between days 8 and 21 of any cycle. If ANC remained below 1000 cells/*μ*L after 28 days, the patient was removed from the study.

Erythropoietin was permitted if a patient's ANC and platelet count exceeded 1500 cells/*μ*L and 100,000 cells/*μ*L, respectively, but the hemoglobin level remained below 8 g/dL and/or a transfusion of packed red blood cells (PRBC) was required. Transfusions of PRBC were given for any hemoglobin level <8 g/dL or for symptoms thought to be related to anemia. Transfusions of platelets were given for any platelet count <20,000/*μ*L or for bleeding associated with a platelet count of <50,000/*μ*L. All blood products were irradiated and leukoreduced.

### Assessment of Toxicity

Toxicity was evaluated using the NCI Common Toxicity Criteria version 3.0 [27]. Serious toxicity events were reported in compliance with the guidelines of the institutional review board, and were reported directly to the Pharmion Corporation Drug Safety Department. Hematologic toxicity, growth factor use, and transfusion requirements were recorded throughout the study. All patients who received azacitidine were included in the safety and efficacy analyses.

## Results

### Patients

Baseline patient characteristics are summarized in Table 1. During the period January 2007 to April 2011, we enrolled 15 patients (9 [60%] males and 6 [40%] females) with AML, median age 74 (range = 64–82) years. At study entry, median BM blast count was 44% (range = 29–92%), and median ECOG PS was 1. Five patients (33%) had high-risk cytogenetics, defined as −5, −7, del (5q), abnormal 3q, or complex karyotype with ≥3 cytogenetic abnormalities. Patients with normal and all other karyotypes apart from *t*(8;21), *t*(15;17), and inv(16) were considered to have intermediate-risk cytogenetics.

**Table 1:** Baseline characteristics.

Patient	Age/Sex	Peripheral blood WBCs/blasts (%)	BM blasts (%)	Cytogenetics at diagnosis
1	70/F	1.8/1	33	High risk
2	64/M	2.4/6	78	Intermediate risk
3	82/M	43.3/38	92	Intermediate risk
4	76/M	3.0/47	31	High risk
5	72/F	1.5/13	49	Intermediate risk
6	74/M	2.5/0	44	High risk
7	82/F	3.1/3	65	High risk
8	77/M	3.2/12	37	Intermediate risk
9	71/F	36.5/7	40	High risk
10	80/F	3.3/4	61	Intermediate risk
11	69/M	1.2/8	81	Intermediate risk
12	77/M	12.6/11	73	Intermediate risk
13	71/M	2.3/0	29	Not available
14	80/F	2.9/7	43	Intermediate risk
15	69/M	1.2/10	33	Intermediate risk

High-risk cytogenetics defined as −5, −7, del (5q), abnormal 3q, or complex karyotype with ≥3 cytogenetic abnormalities; intermediate-risk cytogenetics defined as normal and all other karyotypes except t(8;21), *t*(15;17), and inv(16). BM, bone marrow; F, female; M, male; WBC, white blood cell.

### Response to azacitidine

Using the NCI response criteria, the ORR was found to be 47%. CR, PR, and HI were observed in 20%, 13%, and 13% of patients, respectively (Table 2). Most patients (80%) maintained an ECOG PS of 0–2 during treatment. Four patients (26%) achieved independence from PRBC, while six patients (40%) achieved independence from platelet transfusion. The other pertinent responses to azacitidine are summarized in Table 2, with responses in individual patients being shown in Table 3.

**Table 2:** Overall response to azacitidine.

Variable	
ORR, %	
Total population (*n* = 15)	47
Patients with high-risk cytogenetics (*n* = 5)	40
Patients with intermediate-risk cytogenetics (*n* = 9)	44
Best response achieved in overall patient population, *n* (%)*	
CR	3 (20)
PR	2 (13)
HI	2 (13)
NR	8 (53)
Responses in high-risk cytogenetics group, *n* (%)	
CR	1 (20)
PR	0 (0)
HI	1 (20)
NR	3 (60)
Responses in intermediate-risk cytogenetics group, *n* (%)	
CR	1 (11)
PR	2 (22)
HI	1 (11)
NR	5 (56)
Median treatment duration, days (range)	198 (13–724)
Median time to best response, days (range)	95 (44–279)
Median OS, days (range)	
Total population	355 (13–908)
Patients with high-risk cytogenetics	167 (13–494)
Patients with intermediate-risk cytogenetics	401 (13–908)
Responders to azacitidine	532 (120–908)
Patients maintaining ECOG PS of 0–2 during treatment, *n* (%)	12 (80)
Patients achieving independence from PRBC transfusion while receiving azacitidine, *n* (%)	4 (26)
Patients achieving independence from platelet transfusion while receiving azacitidine, *n* (%)	6 (40)

CR, complete remission; ECOG PS, Eastern Cooperative Oncology Group Performance Status; HI, hematologic improvement; ORR, overall response rate; OS, overall survival; PR, partial remission; PRBC, packed red blood cells.

* Percentages do not total 100 because of rounding.

**Table 3:** Response to azacitidine in individual patients.

Patient	Cytogenetic risk category at diagnosis	Type of response	Time to response (months)	Duration of response (months)	Survival (days) or days alive at LE
1	High	NR	–	–	219
2	Intermediate	NR	–	–	401
3	Intermediate	NR	–	–	13
4	High	NR	–	–	41
5	Intermediate	CR	4	15	908
6	High	HI-P	3	–	–
7	High	CR	2	12	494
8	Intermediate	HI-E, HI-P	3	12	–
9	High	NR	–	–	116
10	Intermediate	PR	1	7	402
11	Intermediate	NR	–	–	120
12	Intermediate	NR	–	–	108
13	Not available	CR	1	–	840 (LE)
14	Intermediate	PR	3	12	509 (LE)
15	Intermediate	NR	–	–	–

High-risk cytogenetics defined as −5, −7, del (5q), abnormal 3q, or complex karyotype with ≥3 cytogenetic abnormalities; intermediate-risk cytogenetics defined as normal and all other karyotypes except *t*(8;21), *t*(15;17), and inv(16). CR, complete remission; HI-E, hematologic improvement-erythroid; HI-P, hematologic improvement-platelet; LE, last encounter; NR, no response; PR, partial response.

**Figure 1:**
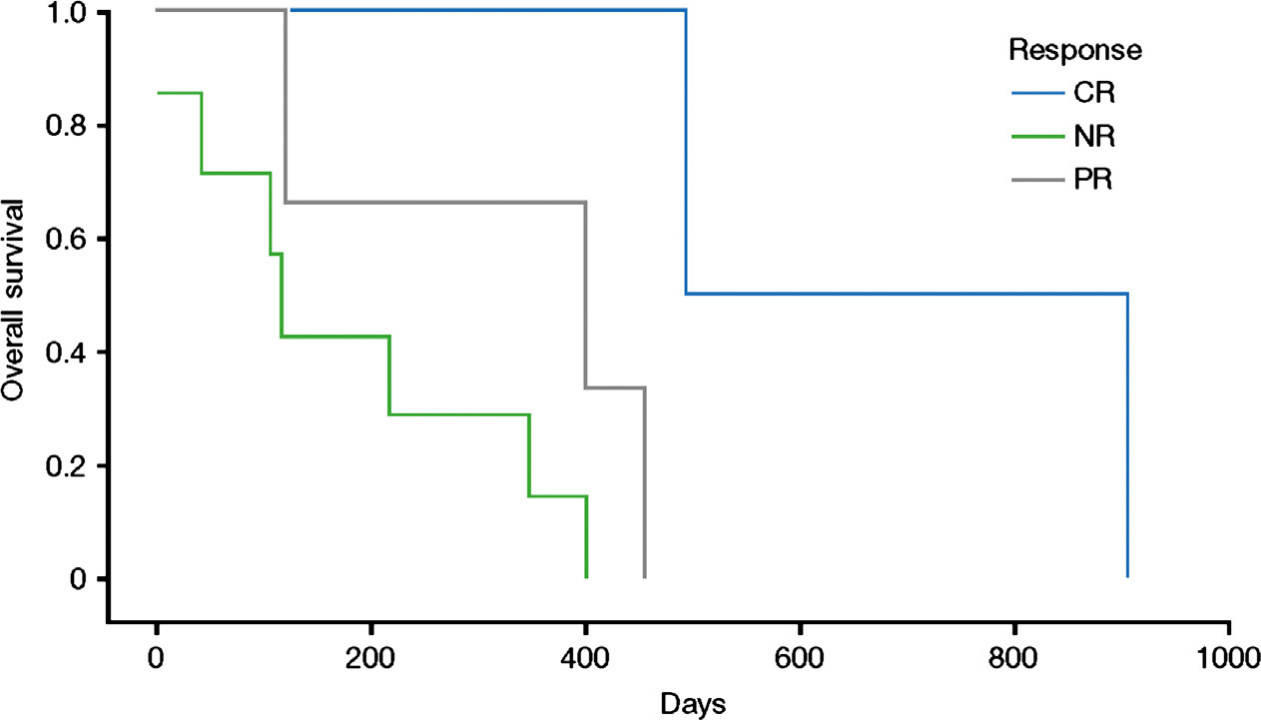
Overall survival by best response to therapy. CR, complete response; NR, no response; PR, partial response.

One patient, who withdrew consent while in CR to be treated closer to home (treatment regimen not available), remained alive at 840 days (time of last encounter), and another patient, who achieved PR, opted to withdraw from the study after 12 cycles secondary to progressive disease (alive at 509 days). Responders had a median BM blast count of 44% (range = 29–65%) at baseline, compared with a median BM blast count of 57% (range = 33–92%) in nonresponders (Table 3). In patients with high-risk cytogenetics (*n* = 5), the ORR was 40%, CR was achieved by one patient (20%), the median OS was 167 days (range = 41–494 days), and the median treatment duration was 5 (range = 2–12) months. In patients with intermediate-risk cytogenetics (*n* = 9), the ORR was 44%, with a CR rate of 11% (*n* = 1), and median OS was 401 days (range = 13–908 days). Median OS was longer in patients with BM blast counts <50% than in those with BM blast counts >50% (364 vs. 260 days). In patients aged ≥70 years (*n* = 12), the ORR was 58% and median OS was 320 days (range = 13–908 days). OS by best response to azacitidine (CR vs. PR vs. No Response) is illustrated in Figure 1.

Of the entire cohort of 15 patients, five patients who failed to either respond or to maintain their initial response to azacitidine while on protocol were deemed suitable candidates for further treatment. Two of these patients received further azacitidine off protocol, while the remaining three received additional treatment with low-dose gemtuzumab ozagamicin. None of these five patients responded to the additional treatment.

### Assessment of toxicity

#### Hematologic toxicity

The most common grade 3–4 hematologic toxicities were febrile neutropenia (40% of patients) and thrombocytopenia (20%) (Table 4). The incidence of hematologic toxicity did not differ significantly between responders and non-responders. The median duration of hospitalization for diagnosis plus treatment of disease-related complications was 19 days (range = 5–56 days), with the majority of therapy being given in the outpatient setting. One patient developed intracranial bleeding from thrombocytopenia (unrelated to treatment), and required prolonged hospitalization. Azacitidine was discontinued in this patient. There were no treatment-related deaths and, of the evaluable patients, most died from either relapse following an initial response or disease progression.

**Table 4:** Grade 3–4 toxicities.

Event	No. of patients, *n* (%)
Febrile neutropenia	6 (40)
Thrombocytopenia	3 (20)
Pneumonia	1 (7)
Diffuse alveolar hemorrhage	1 (7)
Central nervous system bleed (subdural hemorrhage)	1 (7)
Deep-vein thrombosis	1 (7)
Renal failure	1 (7)

The dose and schedule of therapy were adjusted in four patients (27%): one patient required a 25% dose reduction after cycle 3, followed by another 25% reduction after cycle 11, due to drug-induced BM suppression; a second patient required a 25% dose reduction after cycle 2, also due to drug-induced BM suppression; a third patient required and tolerated a 25% dose escalation to recapture CR after cycle 15; and a fourth patient in PR also required a 25% dose escalation after cycle 12 because of progressive disease, but failed to recapture a PR or CR.

### Nonhematologic toxicity

Nonhematologic toxicity was limited to mild injection-site skin reactions and/or fatigue in 73% (11/15) of patients. Other nonhematologic toxicities are as listed in Table 4.

## Discussion

The baseline characteristics of patients in our study were similar to those in other published clinical trials and observations involving the treatment of elderly AML patients with azacitidine [21, 22, 28]. The ORR of 47% in our study is similar to the response obtained in these other studies in patients with a BM blast percentage of 20-30%, in whom azacitidine was infused at the current Food and Drug Administration (FDA)-approved dosage of 75 mg/m^2^/day for 7 days. The documented CR rates in some of these studies ranged from 5% to 23%, and OS was 3–16 months [21, 22, 28]. However, some of the studies did not differentiate between MDS and AML patients, and azacitidine was not necessarily administered in accordance with the FDA-approved dosing schedule. Thus, our findings of an ORR of 47% and a median OS of 355 days (11.8 months) with a 5-day azacitidine regimen in elderly patients with AML suggests similar efficacy to a 7-day regimen.

Earlier trials conducted in elderly patients with AML showed that several factors, either alone or in combination, influence treatment response and survival [29, 30]. These trials suggested that prior therapy, BM blast count, and cytogenetics influence response rates, while OS appeared to be influenced by transfusion dependency, ECOG PS, circulating blasts, and cytogenetics [29, 30]. The French Azacitidine Compassionate Use program (*n* = 138, median age 73 years), which incorporated a 7-day azacitidine dosing regimen, reported an ORR of 21%, a CR rate of 14%, and a median OS of 10.9 months [30]. In our study, median OS and ORR were reduced in patients with high- versus intermediate-risk cytogenetics, and in those with BM blast counts >50% versus those with blast counts <50% (Table 2). In addition, as reported in other published studies [13, 14] the results achieved in patients aged ≥70 years (*n* = 12) were comparable to those in younger patients with a lower BM blast percentage, and to those observed in patients treated with the FDA-recommended azacitidine dosing regimen. Perhaps because of the small number of patients in our study, the results did not meet statistical significance. The small sample size and treatment-naïve status of our subjects limit our ability to draw conclusions about the possible effects of factors such as prior treatments or molecular mutations on the observed outcomes. However, it should be noted that if current azacitidine treatment recommendations were applied to our cohort, many of the patients would have been assigned to best supportive care only. Furthermore, based on studies that have reported a lack of association between response rates, survival, and pretreatment BM blast percentage [21, 28], we suggest that elderly patients with AML who have >30% BM blasts should not be excluded from treatment with azacitidine.

Our study regimen of azacitidine 100 mg/m^2^/day for 5 days was well tolerated, with no treatment-related mortality. Most patients experienced grade 1 toxicity of fatigue and local injection-site reactions that did not require dose reduction or treatment delays. Most grade 3–4 toxic events were cytopenia and associated complications. Neutropenia and thrombocytopenia were the predominant hematologic toxicities, and were managed with transfusion and growth factor support; they did not seem to adversely affect survival. BM suppression requiring dose reduction occurred in only two (13%) patients. One patient underwent dose escalation in an attempt to induce a response, and was able to tolerate the increased dose. Another patient had dose escalation, but failed to recapture the response previously achieved. Febrile neutropenia was the most common cause of hospitalization. At the time of the last evaluation, the most common cause of death in responders was relapse or progressive disease.

There is evidence that varying the azacitidine dosing regimen from the FDA-approved 75 mg/m^2^/day for 7 days does not necessarily affect OS [21, 31]. We hypothesized that a 5-day regimen of azacitidine administered at a dose of 100 mg/m^2^/day, providing a similar cumulative dose to that obtained with 7 days of treatment at 75 mg/m^2^ per day, would be tolerated and similarly efficacious. This hypothesis was shown to be correct. In our study, azacitidine 100 mg/m^2^/day for 5 days was safe and well tolerated, and resulted in a CR rate of 20% and a median OS of 355 days (11.8 months).

In conclusion, a 5-day dosing regimen, offered as outpatient therapy beginning on a Monday and ending on a Friday, potentially offers many practical benefits. We believe that the reduction in visits to the infusion center might translate into cost savings as well as greater convenience for both patients and healthcare providers. Currently, there are very limited data supporting the use of a 5-day azacitidine regimen in elderly patients with AML [21, 28]. However, for practical purposes, we would recommend a regimen of azacitidine 100 mg/m^2^/day for 5 days every 28 days. As previous studies with the standard 7-day azacitidine regimen reported durable responses and survival benefits in newly diagnosed AML patients deemed poor candidates for standard chemotherapy [20, 21], we did not evaluate consolidation treatment in patients who achieved a CR. However, it is possible that consolidation could provide additional benefits in this patient group. Azacitidine administered according to a 5-day schedule and combined with novel agents such as lenolidamide [32], or with histone deacetylase inhibitors such as vorinostat [33] or panobinostat [34], could also potentially produce positive results, and may be worthy of investigation in future studies.
